# Addressing ethnic inequalities in the pathways to care for psychosis

**DOI:** 10.1186/s12916-018-1236-y

**Published:** 2018-12-21

**Authors:** James B. Kirkbride

**Affiliations:** PsyLife Group, Division of Psychiatry, University College London, 6th Floor Maple House, 149 Tottenham Court Road, London, W1T 7NF UK

**Keywords:** Ethnic inequalities, Pathways to care, Psychosis, Psychotic disorder, Mental Health Act

## Abstract

Delays in accessing appropriate care affect patients with most major health conditions, including psychosis. These delays may also be affected by pathways to care. In a recent article in *BMC Medicine*, Bhui and colleagues review the current evidence for ethnic differences in pathways to care for psychosis in England. They reveal that black and Asian people are 3 and 1.5 times more likely, respectively, to come to the attention of psychosis services via compulsory admission than white British people. In this Commentary, I discuss the implications of this on achieving equitable care for psychosis patients and outcomes following their care. The current review of the Mental Health Act provides a timely opportunity to remove such inequalities in England.

Please see related article: https://bmcmedicine.biomedcentral.com/articles/10.1186/s12916-018-1201-9.

## Background

Delays in receiving appropriate, effective treatment for many severe health conditions are associated with a variety of negative sequalae, including adverse effects on morbidity, mortality and quality of life [1]. This is no less true for mental health disorders. Numerous factors contribute to such delays [2], including delays in help seeking treatment and delays in appropriate referral, assessment, diagnosis and treatment. These latter delays arise as a property of the efficiency and capacity within a healthcare system and, for some disorders, as a property of how efficiently that system interfaces with allied professions involved in the care pathway, e.g., social care; police, judicial and penitential services; the charitable sector; educational services; other community stakeholders; or public health practitioners. It stands to reason that the greater the number of stakeholders who have the potential to be involved in the care pathway, the longer the delays in receiving appropriate, effective treatment. In mental health, such issues are exemplified in care pathways for people experiencing psychosis.

### Inequalities in pathways to care

Psychosis describes a set of symptoms principally characterised by false perceptions (hallucinations) and false beliefs (delusions, paranoia). Brief, occasional psychotic symptoms are relatively common in the general population [3] and typically resolve spontaneously for 80% of people [4]. Some people will experience more persistent hallucinations and delusions, accompanied by formal thought disorder, negative symptoms (such as poverty of speech or anhedonia) and cognitive difficulties that meet threshold criteria for a psychotic disorder, such as schizophrenia. The lifetime prevalence of such disorders is estimated to be up to 3% [5]. A longer duration of untreated psychosis is associated with worse clinical, social and functional outcomes for people diagnosed with a psychotic disorder [6] and has provided the rationale for early intervention programs (EIP) in psychosis [7] (and, increasingly, other mental health problems [8]). These programs have gained wide traction in Australia, Denmark, Norway, the UK and increasingly in Asia, Canada and the USA [7, 9, 10]. Although important discussion and empirical research to delineate the optimal intervention length and modes of service configuration is ongoing [8, 11], evidence suggests that when a multimodal EIP of care for young people with psychosis is sustained, this leads to symptomatic recovery, functional recovery and lower mortality rates [10, 12, 13].

The provision of timely access to EIP care – in England, now enshrined in a national policy to commence treatment within 14 days of referral [14] – is consistent with the parity of esteem agenda to bring mental health care standards in line with those in physical health [15]. Nonetheless, these important initiatives to promote equitable access to timely mental healthcare provide only one lens through which differential access to care may arise. Another equally important issue concerns the entrenched inequalities that exist around differential pathways to care for psychosis, as clearly and systematically documented recently by Bhui and colleagues in *BMC Medicine* [16].

In their review, of all primary studies published in the last five years on ethnic differences in pathways to care for psychosis in England, the authors identify 40 studies showing that –relative to the white majority population – people of black Caribbean or African origins were, on average, over 3 times more likely to come to the attention of psychosis services as a result of compulsory admission under the current Mental Health Act (specifically, via civil Section 2 detentions). People from Asian backgrounds were also 1.5 times more likely to come to the attention of services via this pathway. Moreover – and perhaps unsurprisingly – these figures were echoed in police contact and involvement of the criminal justice system in pathways to care. These systemic disadvantages are compounded by the well-established elevated likelihood of developing psychotic disorders experienced by several black and minority ethnic groups compared with the white British population in the UK [17]. Differential pathways to care by ethnicity have also been observed in Canada [18] and the Netherlands [19], though not in New Zealand [20]. This issue has received less attention in the USA, but recent data from the RAISE-ETP trial [21] found similar levels of criminal justice system involvement between black and white participants with first-episode psychosis (4.0% versus 2.3%, respectively; n = 11). However, there were disparities in a variety of other social and clinical characteristics at both first presentation for psychosis [21] and follow-up [22]. It will be important to replicate this finding in larger, first contact population-based samples, given that the the RAISE-ETP trial reported surprisingly low levels of criminal justice system involvement in either group.

The latest data synthesis by Bhui et al. [16] demonstrates that black and ethnic minority groups in England are more likely to be detained against their will, or to arrive at mental health services via the criminal justice system. Given such disruptive and potentially destructive pathways to seeking care for acute psychotic presentations, it is perhaps somewhat surprising that Bhui et al. [16] did not find differences in the duration of untreated psychosis between black and white groups, while such delays were shorter for Asian than white groups. Nonetheless, the available data here came from heterogenous studies and it would be premature to suggest that differing pathways to care did not affect delays in treatment, subsequent care received, or downstream outcomes following the onset of psychosis. Given that all such studies are based on observational data, we also do not know the counterfactual here: had police or judicial services not been involved in pathways to care, what effect would this have had on the duration of untreated psychosis?

## Conclusion

The persistence of ethnic disparities in pathways to care highlighted by Bhui et al. [16] calls for a paradigm shift in our political response to the unacceptable inequalities in pathways to care for psychosis based on ethnicity, race or cultural background. This call is particularly timely in the wake of the British Government’s current review of the Mental Health Act [23] and the recent denouncement by Her Majesty’s Inspectorate of Constabulary and Fire and Rescue Services that the police are increasingly picking up “the pieces of a broken mental health system” [24]. Policymakers have a window of opportunity to sensitively and comprehensively identify and tackle structural and cultural injustices – including the role of institutionalised racism – in the provision of, and access to national mental health services. This need is most apparent for psychosis, but may also extend to other mental health disorders for which pathways to care remain understudied [25]. In the UK we have a long, strong tradition of evidence-based medicine and healthcare, whose values are reflected in the national commissioning of innovative and effective mental health care for psychosis, such as EIP services. With the current review of the Mental Health Act, we now have the opportunity to bring the same standards of evidence to bear on fair and equal access to such services.
